# Asthma and climate change: unveiling hidden vulnerabilities

**DOI:** 10.1007/s11739-025-04176-y

**Published:** 2025-11-23

**Authors:** Francesco Maria Galassi, Elena Varotto, Mauro Vaccarezza, Domenico Ribatti

**Affiliations:** 1 Department of Anthropology, University of Lodz, Łódź, Poland; 2 School of Biomedicine, The University of Adelaide, Adelaide, SA Australia; 3 College of Humanities, Arts and Social Sciences, Flinders University, Adelaide, SA Australia; 4 Curtin Medical School, Faculty of Health Sciences, Curtin University, Bentley, WA Australia; 5 Department of Translational Biomedicine and Neuroscience, University of Bari Medical School, Bari, Italy

**Keywords:** Asthma, Climate change, Air pollution, Pollen, Respiratory mucosa, Child

Climate change is now firmly established as a determinant of respiratory health, with asthma among the conditions most sensitive to its shifting contours [1]. Rising temperatures and elevated atmospheric carbon dioxide drive longer, more intense pollen seasons [2], while extreme events such as wildfires, droughts, and storms degrade air quality. Importantly, air quality and, consequently, respiratory health are also compromised by anthropogenic combustion processes (e.g., fossil fuel burning, vehicular traffic, industrial emissions), which generate pollutants directly responsible for both climate change and respiratory morbidity [3]. The result is a discernible rise in asthma exacerbations, particularly among children and women, whose vulnerability reflects both biological and social factors. This vulnerability is not uniform across the life course: male children have higher asthma prevalence in childhood, whereas after puberty women exhibit higher prevalence and severity (the “sex reversal”), likely mediated by hormonal, immunologic, and gender-related factors [4]. Recent syntheses further underscore these converging risks in the climate–allergy–asthma nexus, which is the interconnected effects of meteorological shifts, allergens, and airway inflammation [5, 16]. Recent analyses have traced clear associations between meteorological instability—oscillations in temperature, extremes of humidity, and erratic wind patterns—and surges in paediatric asthma admissions, highlighting how sudden shifts in climate parameters can precipitate acute respiratory crises [5]. For instance, modelling suggests that climate-related increases in ozone could raise summertime paediatric asthma emergency visits by ~ 7.3% in U.S. settings [6]. Moreover, under a 2 °C warming scenario, approximately 34,500 additional paediatric asthma cases per year are projected globally, approaching ~ 90,000 under higher warming trajectories [7]. The cumulative effect of these exposures weighs most heavily upon children, who possess fewer physiological reserves, narrower airways, and developing lungs that are more readily harmed by air pollutants and aeroallergens, thereby magnifying both frequency and severity of asthma exacerbations in this vulnerable group. Alongside epidemiological insights, there is scope for methodological renewal. Precision-cut lung slices (PCLS), air–liquid interface models (ALI), and lung-on-a-chip systems offer novel means of interrogating how pollutants and bioaerosols, modulated by climate change, interact with airway epithelia [2]. PCLS preserve the structural and multicellular complexity of human lung tissue, enabling ex vivo modelling of airway physiology and injury–repair dynamics [9]. ALI cultures allow primary airway epithelium to differentiate and recapitulate in vivo-like exposure to airborne stimuli [10]. Lung-on-a-chip platforms reproduce key features of the pulmonary microenvironment under dynamic flow, supporting mechanistic studies of pollutant and allergen effects [11]. While powerful, their routine use remains limited by cost, technical complexity, and the need for specialised infrastructure. There is, however, a further dimension that has attracted scant notice. Beyond heat and allergens lies the insidious role of climate-amplified chemical pollutants in undermining epithelial barrier integrity. Airborne particulates, volatile organic compounds, phthalates, and polycyclic aromatic hydrocarbons have each been implicated in erosion of mucosal defences [2, 5]. Equally important are gaseous pollutants such as nitrogen dioxide (NO₂), sulfur dioxide (SO₂), carbon monoxide (CO), and ozone (O₃), all associated with asthma incidence and exacerbations [3]. The “epithelial barrier hypothesis”, here applied to paediatric asthma in the context of climate change, posits that environmental stressors disrupt epithelial integrity, increase permeability, and promote chronic inflammation, a framework that now warrants systematic mechanistic and epidemiological investigation to elucidate how pollutants, aeroallergens, and climate-related extremes converge on barrier dysfunction [12]. In conclusion, strategies to mitigate climate-related asthma risk must extend beyond allergen calendars and emergency preparedness. Primary prevention measures include exclusive breastfeeding, associated with reduced risk of wheezing and asthma in predisposed children [13]; comprehensive home-based interventions to reduce allergens, dampness, and mould [14]; and rural/ “farm-like” lifestyle exposures that enhance early-life microbial diversity and appear protective against allergic sensitisation and asthma [15]. At the population level, transitions to clean energy, reductions in fossil-fuel combustion, and urban greening with low-allergenic vegetation offer co-benefits for climate mitigation and respiratory health [3, 16]. Research integrating barrier biology with advanced in vitro modelling could illuminate critical early-life tipping points where preventive interventions might prove most effective. Policy frameworks, likewise, should reckon not only with the obvious meteorological threats, but also with the more subtle barrier-compromising pollutants whose influence is magnified by our changing climate.
